# Miniature bioreactors: current practices and future opportunities

**DOI:** 10.1186/1475-2859-5-21

**Published:** 2006-05-25

**Authors:** Jonathan I Betts, Frank Baganz

**Affiliations:** 1 The Advanced Centre for Biochemical Engineering, Department of Biochemical Engineering, University College London, Torrington Place, London, WC1E 7JE, UK

## Abstract

This review focuses on the emerging field of miniature bioreactors (MBRs), and examines the way in which they are used to speed up many areas of bioprocessing. MBRs aim to achieve this acceleration as a result of their inherent high-throughput capability, which results from their ability to perform many cell cultivations in parallel. There are several applications for MBRs, ranging from media development and strain improvement to process optimisation. The potential of MBRs for use in these applications will be explained in detail in this review. MBRs are currently based on several existing bioreactor platforms such as shaken devices, stirred-tank reactors and bubble columns. This review will present the advantages and disadvantages of each design together with an appraisal of prototype and commercialised devices developed for parallel operation. Finally we will discuss how MBRs can be used in conjunction with automated robotic systems and other miniature process units to deliver a fully-integrated, high-throughput (HT) solution for cell cultivation process development.

## Review

### Introduction

The advent of molecular biology and genetic-manipulation technology over the last quarter of a century has had a dramatic effect upon the pharmaceutical/healthcare industries, with a large number of the many applications of this technology being based on the ability to create recombinant cell lines for human therapeutic benefit [1,2]. In addition to the development of these genetically-modified organisms, there remains a need to improve wild-type productivity, accelerate the screening of newly-discovered microbes and continue the progression of related tasks such as growth medium improvement and process optimisation. Traditionally, cell cultivation process development has required the screening of large numbers of cell lines in shake flask cultures, and from this the further testing of successful candidates in bench-top bioreactors prior to pilot-scale studies [3]. The need to carry out a vast number of development cultivations has resulted in the advance and increasingly widespread deployment of small-scale bioreactor systems that offer a miniaturised, HT solution to process development.

The main cell types used to produce therapeutic products are bacterial and mammalian cells, each of which possesses unique benefits and limitations that influence the type of bioreactor used for process development. Bacterial cells are generally robust and not susceptible to shear damage, meaning that highly-shearing radial impeller systems (e.g. Rushton turbines) and high agitation rates can be employed. This provides such bioreactors with a high mass transfer capability, allowing rapidly metabolising, high-cell density microbial cell cultivations to be supported and increasing the amount of product that such bioprocesses can yield. Although mammalian cells don't have a protective cell wall and so are typically more shear-susceptible and require gentler handling than their bacterial counterparts, most of the commercially-used cell lines can be grown in stirred tank bioreactors, albeit with design modifications. For example, low-shear, marine-type axial impellers can be used instead of Rushton turbines to gently circulate the cells and nutrients in a baffle-free environment; and shear protectants such as serum or Pluronic F-68 can be added to cell culture media [4].

In addition to therapeutic drug development, MBRs can be used for growth medium development; strain improvement through metabolic engineering or directed evolution; and so-called bio prospecting of natural products – all of which are processes that carry a large bioreactor burden which can be alleviated by the use of HT miniature devices. In particular, MBRs can reduce the labour intensity and materials cost of the vast number of cell cultivations necessary in bioprocess development, increasing the level of parallelism and throughput achievable, and as such are of growing interest [5-7]. It is important that such devices when used for process development can be relied upon to accurately mimic laboratory and pilot scale bioreactors so that growth kinetics and product expression – optimised at miniature scale – can be expected to scale-up quantitatively.

**Figure 1:**
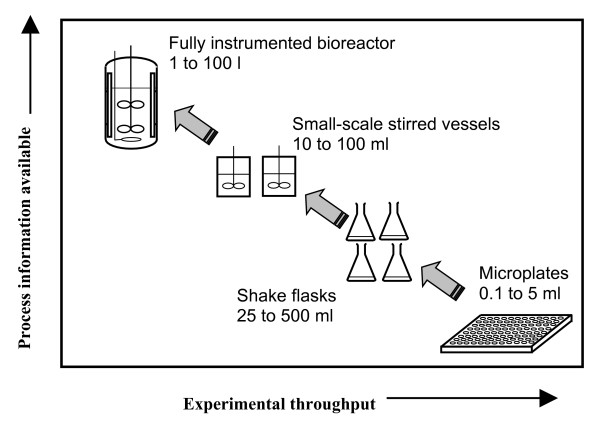
Illustration of the trade off in information output versus HT capability that currently exists for various cell cultivation devices at different scales (adapted from Doig et al., 2006 [3]). This figure shows that as bioreactors increase in scale, typically more process information is available due to improved monitoring and control systems.

Whilst undoubtedly being more capable of HT operation than conventional, laboratory-scale bioreactors, MBRs typically are currently less instrumented and also have limited opportunity for off-line sampling due to the small volumes used (ranging from ca. 0.1 ml to approx. 100 ml); this means that there is currently a trade-off between information content in terms of data quality and quantity available from the bioreactor obtained by both online and off-line measurement and experimental throughput, illustrated in Figure 1. As no device has yet solved all of the challenges of miniaturising, i.e. accurately mimicking large-scale process conditions and yet still retaining the full functionality of conventional bioreactors, it is the intention of the authors to review current developments and then indicate where the technology is likely to progress in the future so that the current HT benefits can be expanded and the information gap that currently exists between miniature and lab-scale bioreactor platforms is reduced. This review has grouped the various MBRs described on the basis of their agitation method (i.e. shaking, stirring or gas-sparging) with reference to the type of conventional bioreactor they either mimic or are derived from; the key specifications and characteristics of prototype and commercialised miniature cell cultivation devices capable of parallel operation are summarised in Table 1.

**Table 1:** A comparison of the miniature bioreactor systems (MBRs) that have been reported to be capable of parallel operation illustrating key technical and performance specifications.

**Device/Reference**	**Based on**	**Volume (ml)**	**Agitation/Aeration**	**pH, DOT and OD instrumentation**	**KLa (h^-1^)**	**Method**	**Multiplexing**
Fedbatch-Pro, **	Shake flask	50 – 500	Orbital shaker; Surface	pH via sterilisable probe	N/R	N/A	16
MicroReactor, **	MTP + microfabrication	3–5	Orbital shaker (up to 800 rpm); Gas sparging	pH and DOT via optical probes	N/R	N/A	24
SimCell, **	Microfluidic chip	0.3 – 0.7	Rotation of MBR chips; Surface via membrane	pH, DOT and OD at-line via cell-reading station	Up to 500	CFD estimation	1500
MBR array [59]	MTP + microfabrication	0.25	Orbital shaker (175 rpm); electrochemical O_2 _generation	pH (ISFET sensor) and OD optically	Unknown	N/A	8
Polymer-MBR [41, 56]	Microfabrication	0.15	Magnetic stirrer bar (200–800 rpm), Surface via membrane	pH, DOT and OD via optical probes	20 – 75	Dynamic gassing-out	8
Stirrer-Pro flask, **	STR	200 – 275	Magnetic stirrer bar (10 – 1000 rpm); Sparger	pH and DOT via sterilisable probes	N/R	N/A	16
Xplorer, **	STR	Up to 100	Single turbine impeller (100 – 2000 rpm); Sparger	pH, DOT and OD probes	400	Dynamic gassing-out	16
Cellstation, **	STR	Up to 35	Dual paddle impeller (10 – 1000 rpm); Sparger	pH, DOT and OD via optical probes	N/R	N/A	12
MSBR [27, 40]	STR	18	Triple turbine impeller (up to 7000 rpm); Sparger	pH and DOT via optical probes	Up to 480	Dynamic gassing-out	N/R
Bioreactor block [39, 44]	STR	8 – 12	Gas-inducing single impeller (up to 4000 rpm)	DOT optically; pH and OD via plate reader	700 – 1600	Dynamic gassing-out	48
Parallel BCR [52, 53]	Bubble column	200	Gas-sparging	pH and DOT probes	Up to 540	Dynamic gassing-out	16
MBCR [49, 51]	Bubble column	2	Gas-sparging	pH and DOT via optical probes	Up to 220	Dynamic gassing-out	48

Nomenclature: SF = shake flask, MTP = microtitre plate, MSBR = miniature stirred bioreactor, STR = stirred tank reactor, MBCR = miniature bubble column reactor, DOT = dissolved oxygen, OD = optical density, ISFET = ion-selective field effect transistor, N/A = not applicable, N/R = not reported.

### Miniature shaken bioreactor systems

Shaken systems have been used in bioprocessing from the very first attempts to grow antibiotic-producing microbial cultures in the 1940s. They are still widely used in industry and academia as a tool for drug discovery; media, strain and product optimisation; and process development [8-11]. They comprise many different designs and volumes, ranging from shake flasks of hundreds of millilitres right down to microtitre plates (MTPs) of a few microlitres in volume.

#### Shake flasks

For the past fifty years, scientists have used cell cultivation in shake flasks as a means of process development on a small scale, with volumes ranging from ca. 10 ml to 500 ml [12]. Shake flasks come in a variety of guises, can be made out of glass or plastic and some have baffles to aid aeration and mixing. They can be agitated using either orbital or linear shaking and can be housed in a temperature-controlled cabinet. Factors that affect shake flask cultivations include vessel size, fill volume, construction material, geometry of baffles, shaking frequency and type of plug used to seal the vessel. Büchs [12] asserts that shake flasks have been estimated to be used for over 90% of all culture experiments across industry and academia, growing a wide range of microorganisms e.g. bacteria [13], fungi [14], and yeasts [15] as well as mammalian cells [8]. It is easy to see why they are so widely used: they are an inexpensive and effective way of reproducibly performing many types of industrially-relevant cell cultivations for process development. Moreover they are easy to operate and largely impervious to mechanical complications. Throughout most of the long period of their use there was little significant modification of the technology [16], with no online monitoring of cultures and manual additions and sampling. Only recently has there been the introduction of instrumented shake flasks, designed to measure and potentially control pH and DOT levels online [15,17]. pH and dissolved oxygen can be measured using a ruthenium oxide dye that quantifiably fluoresces in the presence of hydrogen ions or oxygen respectively when excited with an LED lamp. This dye can either be incorporated into a patch and adhered inside a flask or coated onto the tip of a fibre optic-linked probe and immersed into the culture of interest. Other parameters that can now be measured online include oxygen transfer rate (OTR) and carbon dioxide evolution rate (CER) – and from these the respiratory quotient (RQ) can be derived [7]. Having such parameters monitored online would allow for more sophisticated cell-cultivation strategies to be carried out such as substrate feeding based on changes in culture broth pH due to cell metabolism [18]. Furthermore, Akgün *et al*. [11] have recently developed a novel shake flask system that is capable of continuous operation and thereby increases the scope of parallel bioprocess development using shaken systems.

However, a major limitation of shake flasks is their reliance on surface aeration, leading to reduced oxygen transfer relative to stirred tank reactors (STRs). Wittmann *et al*. [17] reported overall volumetric mass transfer coefficient (k_L_a) values of up to 150 h^-1 ^in shake flasks. k_L_a values of 151 h^-1 ^(600 ml, 200 rpm) to 277 h^-1 ^(100 ml, 200 rpm) have been recorded in a novel, box-shaped, shake flask system developed by Kato and Tanaka [9], which are sufficiently high to perform most batch cell cultivations without inhibiting microbial growth. These researchers incorporated gas-permeable membranes in the upper corners of their prototype flasks which allowed for more effective gas flow into the vessel during shaking, overcoming the problem found in conventional shake flasks of introducing more air into the system in a sterile way. For the purpose of cultivations where the oxygen demand is high, the introduction of baffles can increase OTR at lower shaking frequencies [19]; however, high speeds can lead to excess splashing which can cause the gas-permeable plug (often made of cotton wool) at the top of the flask to become blocked through liquid saturation. Such an obstruction has been shown to severely reduce the oxygen transfer capability of the system, which could cause problems if a rapidly-respiring aerobe was being grown [20]. Oxygen starvation could slow down the growth rate, alter production formation rates and/or generate unwanted toxic by-products e.g. acetate formation by *Escherichia coli *[21].

#### Microtitre plates

MTPs (also called microwell plates) were first introduced in 1951 as a platform for diagnostic tests and are still widely used in the life sciences [22]. They handle diagnostic tests such as enzyme-linked immunosorbent assays that take advantage of the ability to perform many identical reactions in parallel and at a very small scale. It is this advantage that has led to MTPs being used as miniature shaken bioreactors in the screening stage of process development for cell-line evaluation [23]. Plates are usually fashioned out of plastic, although glass and metal versions exist. Mixing can be achieved using pipette aspiration or magnetically-agitated stirrer bars; however, orbital shaking of the entire plate on a heated block capable of controlling culture temperature is by far the most common method. The number of wells contained in MTPs is typically 6, 12, 24, 96 and 384, with up to 1536 and 3456 wells now available for ultra high-throughput screening (UHTS) [24]. Wells can either be rectangular or cylindrical, with square geometries aiding mixing and oxygen transfer by mimicking the action of baffles. Square-bottomed plates act in a similar way by limiting vortexing of liquid inside the well and thus increasing the turbulence of the system. Due to the increase in surface area caused by greater fluid dissipation up the sides of each microwell and the increased driving force for oxygen caused by better mixing, OTR is proportional to shaking amplitude and frequency, therefore increasing these parameters can be beneficial [23]. In addition, Hermann *et al*. have reported OTR to be inversely proportional to fill volume, particularly at higher shaking frequencies [25]. However, there is a point beyond which any increase in agitation results in spillage of process liquid (unless the well is capped – which has it own problems, with reduced oxygen transfer into the well). As with shake flasks, the relatively low oxygen-transfer capacity of MTPs (k_L_a values of up to 200 h^-1 ^in 96 well plates) stems from the fact that they are shaken systems and rely upon surface aeration for mass transfer. In contrast, Kensey *et al*. [26] reported k_L_a values using the sulfite oxidation method of up to 1600 h^-1 ^in a 48-well, standard geometry MTP with 3 mm orbital throw at 1400 rpm using a filling volume of 300 μl, which is comparable with conventional STRs. By using a calculated proportionality constant, this team were able to relate the oxygen transfer capacity obtained using a chemical method to biological media.

There are also methods available for determining k_L_a at small-scale which provide data that are directly comparable with values obtained under process conditions. For example Duetz *et al*. [23] and Doig *et al*. [49] estimated k_L_a by mass balance under conditions of oxygen limitation from the linear growth of *Pseudomonas putida *in an MTP and *Bacillus subtilis *in a prototype miniature bubble column reactor (MBCR) respectively. In addition, the dynamic gassing-out method is often preferable to the sulfite oxidation method for the determination of k_L_a values as it is usually carried out in water [27]. Consequently this system is coalescing and, whilst not identical to biological media, it is more representative of cell cultivation conditions than the totally non-coalescing conditions of the sodium sulfite method. However, this technique is difficult to use in MTPs as shaking often has to be stopped before DOT measurement in order to get accurate readings, thus altering the mass transfer environment at a critical moment. Due to the problems associated with using established methods for k_L_a determination in MTPs we have recently developed a novel method that is based on the bio-oxidation of catechol by the enzyme catechol-2,3-dioxygenase [28]. This method yielded similar k_L_a values compared to the dynamic gassing-out method and since it is rapid and doesn't require any assumptions about the kinetics we believe that this method is well suited for k_L_a evaluation in MTPs and other small-scale devices.

MTPs also suffer to a degree from the very feature that makes them attractive as a high-throughput device – small volumes – because evaporation can remove a significant proportion of the fluid in the well [7]. Breathable membranes can be placed on top of the plates to limit this evaporation, yet then the oxygen transfer capabilities are reduced. Zimmermann *et al*. [29] reported on a membrane that achieved a moderate degree of water retention and oxygen transfer; however, k_L_a values were reduced by a factor of five, further exacerbating the problem of low oxygen transfer capability inherent in shaken systems. Although evaporation is a potential problem in all MBRs, MTPs appear to be more susceptible to this due to typically using the smallest process volumes. MTPs of 3456 wells offer the highest throughput of any miniature cell cultivation device available, and have been shown quantitatively to sustain growth of Chinese Hamster Ovary (CHO) cells [24], although such a miniscule process volume (1 – 2.2 μl) means this device would probably not be able to mimic the mechanisms by which larger shaken vessels operate; for example surface tension effects would extend throughout the well, severely limiting mixing capability. Furthermore, no removal of medium for off-line sampling would be possible.

Although MTPs are used extensively in discovery research they have suffered from a lack of instrumentation in a similar way to shake flasks, limiting the range of data that can be collected. However, recently techniques have been developed to measure pH and DOT in such systems [30-32]. For example Lye and colleagues have studied the effect of pH control on biomass yields and growth kinetics of a filamentous bacterium in an MTP [33]. Despite some of the inherent limitations of MTPs when performing cell cultivations, progress has been made in the characterisation of mixing, mass-transfer and instrumentation of these vessels, meaning that the unique advantages of these devices in terms of automation potential and intrinsic HT capability are leading to their growing use as early-stage MBRs.

#### Spin tubes

Early-stage small-scale mammalian cell culture process development has traditionally been carried out in T flasks and small-scale bioreactors (often spinner flasks, typically of 500 ml volume) [34,35]. Although initially largely undefined devices, work has been performed to characterise the engineering environment in spinner flasks which has made them easier to use as scale-down vessels [36]. Nevertheless, the fact remains that their relatively large volume makes them non-viable as a HT technology, meaning that there is a real requirement for miniature bioreactors to be used in conjunction with mammalian cells for parallel cell cultivations. Recently spin tubes have been developed and used as a small-scale process development tool for cultivation of mammalian cells. The spin tubes first described by De Jesus *et al*. [37] appear to offer several advantages over spinner flasks, such as smaller process volume. They have since been commercialised by ExcellGene SA (Valais, Switzerland) under the name TubeSpin Satellites. These culture vessels consist of modified 50 ml centrifugation tubes mounted on a rotating orbital shaker placed in an incubator. Culture volumes are 5 ml to 35 ml per reactor and off-line analysis is carried out using entire tubes on a sacrificial basis. This system does not have the instrumentation necessary to carry out fully characterised mammalian cell cultivations; however, it is a useful tool for media optimisation and productivity enhancement and gives cell culture development a high-throughput aspect, with the developers of this system reporting the ability to process 1000 different cultures per week. The relatively large volume and low evaporation rates found in this device are assets when dealing with slow-growing mammalian cells, where cultures can be many days in duration, yet it should be pointed out that no engineering characterisation of mixing and mass transfer has been carried out in this system and thus spin tubes are largely used for screening applications.

### Miniature stirred bioreactor systems

**Figure 2:**
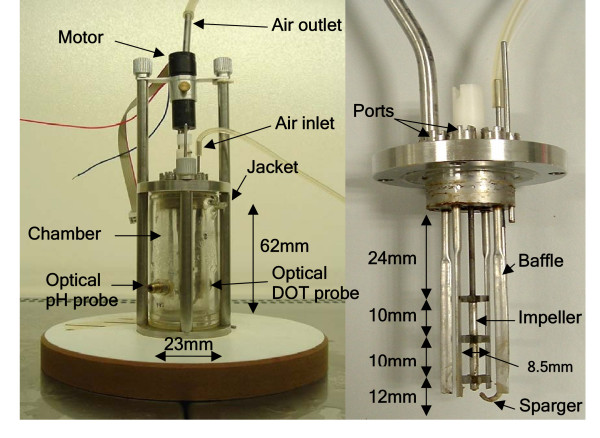
Technical illustration of an 18 ml working volume miniature stirred bioreactor (MSBR) prototype [40].

Miniature stirred bioreactors (MSBRs) based on conventional STRs have been developed as an alternative to shaken MBR systems for early-stage process development and cell characterisation. Typically these devices are closely modelled on lab-scale bioreactors and thus permit greater potential for monitoring and control than other miniature bioreactor platforms. They are usually of a process volume intermediate between MTPs and shake flasks [38,39] and construction materials vary widely, with Perspex [38], Pyrex [40], poly-methylmethacrylate (PMMA) [39,41] and stainless steel [40,42] all being used. Figure 2 illustrates our 18 ml working volume prototype MSBR that is constructed of stainless steel and Pyrex and equipped with optical probes to measure pH and DOT online. This vessel has been characterised in terms of its mixing efficiency and oxygen transfer capability [40]. It has been shown to be capable of mimicking conventional STRs in cell cultivations of varying rheology, shear-sensitivity and oxygen demand (i.e. the filamentous bacterium *Saccharopolyspora erythraea *producing erythromycin and recombinant *E. coli *producing plasmid DNA and antibody fragment respectively [40]). The device could successfully grow a range of organisms due to its relatively high k_L_a values (480 h^-1 ^at 7000 rpm using the dynamic gassing-out method) and short mixing times (4.8 s at 7000 rpm – over twice as fast as a 7 L vessel based on equal specific power input). High oxygen transfer rates supported growth of quickly respiring organisms (*E. coli*), whereas effective mixing allowed the vessel to maintain homogeneous conditions when dealing with viscous fermentation broths – often found when growing filamentous organisms. The agitation rate could also be very tightly controlled, which helped to prevent damage to shear-sensitive mycelial organisms through excessive power input. In addition, the gassed power consumption of the vessel has been measured, resulting in the calculation of the impeller power number over a wide range of operating conditions and therefore allowing cell cultivations to be reliably scaled down on the basis of equal specific power input [27]. Although this MSBR is a prototype, it would be possible to multiplex such a device in order to obtain a higher throughput.

By providing agitation and actively aerating the vessel, mass transfer rates close to a conventional, laboratory-scale STR have been reported for other MSBRs in the literature. For example, Lamping *et al*. [38] reported k_L_a values of 360 h^-1 ^at 1 VVM and 3000 rpm using the dynamic gassing-out method in a prototype MSBR similar in design to that shown in Figure 2. In addition, the same team successfully modelled oxygen transfer in a prototype miniature bioreactor using computational fluid dynamics (CFD) analysis, which was based upon the relevant engineering parameters of the velocity field, bubble size, gas hold-up and the energy dissipation rates inside the MBR [38].

Puskeiler *et al*. [39] recently reported k_L_a values of over 700 h^-1 ^(12 ml volume) and as high as 1600 h^-1 ^(8 ml volume) for an MSBR agitated at 2300 rpm. This system uses a novel gas-inducing impeller that results in a very high oxygen transfer capability. In that study the dynamic gassing-out method was employed to measure k_L_a, though non-coalescing conditions were used, making direct comparison with values from cell-cultivation media or coalescing fluids difficult. In the same paper the ability of the system to sustain fed-batch cell cultivations was described, which illustrates the potential of miniature bioreactor technologies to support such industrially-important strategies. In addition, the feasibility for online monitoring and control was shown. The device described in that report, designed in association with H+P Labortechnik AG (Oberschleissheim, Germany) is an integrated unit ("Bioreactor Block") capable of supporting up 48 cell-cultivations simultaneously [6,43]. An integrated liquid handling system enabled pH to be measured at-line with a frequency of one hour by dispensing samples of 20 μl into commercially-available MTPs containing affixed pH patches. Eight minutes later the same liquid sampling system then adjusted the pH using 4 M NaOH. Whilst the use of automated liquid handling to control pH is a neat solution, the authors acknowledged that this may be impractical if used with sensitive organisms that require more responsive pH adjustment. However, the report states that an improved monitoring system is being developed with industrial partners to provide more frequent monitoring which may increase the number of simultaneous fermentations able to be effectively monitored. DOT was measured in the system using a prototype sensor block with optical probes, though only 8 reactors of the 48 cultivation vessels were monitored simultaneously [44]. Such a device can also be integrated with standard robotic equipment to perform liquid-handling tasks such as inoculation, feeding and sampling [6].

Using a different approach Fluorometrix Corporation (Stow, Massachusetts, USA) has developed a multiple-vessel MSBR construct called Cellstation^®^. This MBR uses optical technology to permit *in situ *on-line monitoring of up to 12 parallel cultivations for pH [45], DOT [46] and optical density (OD) and agitation is provided by dual paddle-type impellers. Each vessel has a working volume of up to 35 ml and is attached to a carousel which rotates allowing all vessels to be sampled and monitored sequentially. The optical sensor system has been validated by showing the consistency of the pH and DO sensors over a period of 70 hours in a mammalian cell culture process [47]. In addition, Rao's research group at the University of Maryland which has close links with the company has recently published details of two prototype 24-well MSBR systems that further improve the throughput of this technology [48].

In parallel with these MSBR developments Dasgip AG (Jülich, Germany) have introduced the Stirrer-Pro Flask, part of their Fedbatch-Pro^® ^cell-cultivation series, which comprises up to 16 culture vessels (working volume 200–275 ml) and offers an agitation-driven oxygen transfer capacity, and a fed-batch capability. pH and DOT can be monitored using standard sterilisable probes and controlled independently for each vessel by automatic acid/base liquid additions and air-flow rate/agitation variation respectively. Substrate addition can be linked to either DOT or pH trigger points allowing a fully-automated fed-batch capability. The combination of mechanical agitation (between 10 – 1000 rpm) and gas sparging indicates that this system is capable of supporting fast-growing bacterial cultures to a high cell density and therefore would be useful in the development of such bioprocesses. However, the working volume used is relatively large compared to most of the other systems discussed and the set-up is complicated by the presence of a large number of tubes and wires for additions and measurements. A variant of this system containing up to 16 shake flasks equipped with pH probes has also been developed allowing intermittent feeding and parallel pH control [6].

As a smaller alternative to lab-scale STRs capable of parallel operation such as the Sixfors^® ^system developed by Infors AG (Bottmingen, Switzerland), researchers at University College London, in association with HEL Group's BioXplore bioreactor business (Barnet, UK) have developed and characterised a 4 – 16 chamber MBR system with fully integrated and automated control of DOT and pH. Although each vessel has a maximum working volume of 100 ml, thereby being towards the upper end of MSBR technology, the development of stand-alone software to monitor such bioreactors is a step towards endowing MBRs with the same degree of control and automation that exists with conventional bioreactors.

### Miniature bubble column reactors

Bubble columns utilise gas sparging instead of agitation as a means of promoting mixing and oxygen mass transfer for cell cultivation. As an alternative to stirred or shaken devices we have developed a miniature bubble column reactor (MBCR) that is based on an MTP with porous membranes (frits) acting as the entire base to each individual well [49]. Air permeates the frit and flows up through each well, providing oxygen for each growing culture. Provided that each frit is manufactured to a high specification and has an identical degree of porosity, the flow rate to each column is equal and can be calculated. This prevents air-flow rate variance artificially affecting results.

Doig *et al*. [49] detail the construction and characterisation of a prototype 12-well MBCR that is capable of supporting the aerobic cultivation of *Bacillus subtilis *cultures with each column having a working volume of 2 ml. k_L_a values were reported up to 220 h^-1 ^using the dynamic gassing-out method at a superficial gas velocity of 0.02 ms^-1^. One of the benefits of this type of device is that, unlike an MTP, aeration is via direct sparging. This has the effect of increasing the oxygen mass transfer capability of the system relative to an MTP because sparging increases the surface area available for gas-liquid mass transfer relative to surface aeration alone. Although some k_L_a data for MTPs detailed in this review are substantially higher than the MBCR values measured, it must be pointed out that many of the MTP values were derived under rather artificial conditions designed to maximise oxygen transfer, whereas the k_L_a values for the MBCR shown above would be reproducible under cell cultivation conditions.

**Figure 3:**
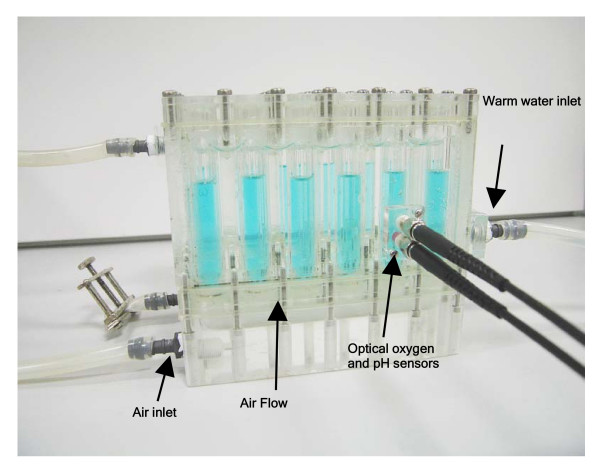
Diagram of the miniature bubble column reactor (MBCR) prototype designed and developed at UCL.

In addition to a large surface area available for oxygen transfer, the lack of agitation in MBCRs means that power input, and therefore oxygen transfer is easier to model than in STRs as there are fewer parameters to consider, with superficial gas velocity and bubble size distribution being key parameters in scale-up/scale-down of bubble columns [50]. Furthermore, the device is stationary, as opposed to shaken, which allows for easier instrumentation as agitation of most MTP systems has to be stopped before measurement in a plate reader can take place. The mechanical simplicity coupled with potentially high oxygen transfer and ease of sampling makes MBCRs suitable for parallel cell cultivation. This could be for the purpose of medium or strain improvement, and early-stage process development, among others. MBCRs could also be used to mimic and predict the performance of large-scale reactors. In this respect we have recently demonstrated a good correlation of oxygen transfer rate with volumetric power consumption (P/V) for miniature (2 ml) and laboratory-scale (100 ml) bubble columns using gas diffusers with the same pore size that allows the prediction of k_L_a as function of P/V [51]. In the same work we also showed a comparable cell cultivation performance using the MBCR relative to a laboratory-scale STR based on equal k_L_a values. These results indicate the potential of the MBCR as a scale-down device. This prototype MBCR device was not instrumented, although in subsequent work we have equipped this device with optical fluorescence patches and used it to measure DOT during cell cultivations. Temperature was able to be controlled by linking the device to a water bath and circulating temperature-controlled water through the enclosed space between the columns (see Figure 3). Similar MBCRs have been developed previously by others [52,53]; however, these vessels use volumes of ca. 200 ml and are therefore two orders of magnitude larger than the device described by Doig *et al*. [49], limiting the degree of parallel operation achievable.

### Other miniature devices

Utilising the concept of an integrated sensor plate, MicroReactor Technologies (Mountain View, CA, USA) have developed a hybrid cell cultivation system based on a shaken, baffled 24-well MTP with a well configuration that allows uniform heat transfer across the plate. The suggested working volume of each well range from 3 to 5 ml and air is introduced to the liquid phase by sparging through sinters located in the base of each well, boosting oxygen transfer capability compared to similarly-designed shaken systems. This recently commercialised cultivation device (licensed in Europe by Applikon Biotechnology AB, Netherlands) is instrumented using fibre optics probes to monitor DOT and pH online in all wells simultaneously. The device also allows independent control of temperature, DOT, pH (via gas sparging) and air flow-rate for all 24 wells. The device overcomes one of the fundamental problems when dealing with MTP-based HT devices – namely how to fit instrumentation to all wells contained within the plate – by attaching all sensor patches to the base of each well and then placing the entire plate onto a shaking incubator platform that has integrated instrumentation circuitry, thereby allowing each well to be independently monitored. The main application is likely to be for the early stages of process development (e.g. strain selection and medium optimization). No data is publicly available yet on engineering characterization of mixing and oxygen transfer and comparison of cultivation performance with lab-scale bioreactor data.

There have been recent developments aiming to reduce the scale of MBRs to sub-millilitre process volumes. Although these miniature systems offer the greatest scope for HT application, there is a practical limit to how small culture volumes can become. Devices that utilise too small a process volume may find it unfeasible to perform cultivations with sufficient monitoring and sampling. Although OD, DOT and pH are able to be monitored online, other critical parameters such as substrate concentration and product yields are frequently not; however, it may be possible to circumvent this problem for certain processes by incorporating markers such as green fluorescent protein into the product [30,54]. Evaporation may become a significant problem in such extremely small culture volumes if working with long bacterial and mammalian cell cultivation processes; also, given the extremely small process volume it would be technically challenging to accurately control the pH through liquid addition. Nevertheless, the scale of operation represents a radical advance in the design of MBRs and significantly increases their potential use for HT parallel cell cultivation.

In this respect, Jensen's research group at MIT have developed a sub-millilitre MBR prototype [55] that has been modified [56] and extended to a multiplexed system capable of carrying out eight instrumented micro-cell cultivations with working volumes of 150 μl [41]. Using standard microfabrication methods, the cultivation wells made of PMMA and poly(dimethylsiloxane) (PDMS) are immobilised on an aluminium base containing all sensor elements and oxygen transfer is enabled via diffusion through a gas-permeable membrane and magnetic stirrers capable of controlling agitation individually to each reactor respectively. DOT, pH and OD can be monitored online using optical probes. The group reported that the device can sustain *E. coli *batch cultivations, yet DOT fell to 0% after 2–3 hours, possibly resulting in oxygen limitation [41]. This is likely considering that the max. k_L_a value measured in this MBR was only 75 h^-1^. Nevertheless, the authors demonstrated that growth behaviour was comparable with that obtained using a range of larger cell cultivation devices [56]. The same research group also detail DNA microarray gene expression analysis of *E. coli *grown in a 50 μl MBR [57]. This work marks a real advance in MBR development as it not only shows proof of principle, but also permits highly parallel analysis of gene expression and could be used to improve understanding of cell physiology during cultivation using a systems-level approach [58]. Maharbiz *et al*. [59] reported the development of an array-based device combining microwell reactors with silicon microfabrication technology that is capable of supporting *E. coli *cultivation in eight 250 μl wells simultaneously. Similar to the MIT reactor (described above) the wells were situated on a base plate containing sensors for pH and OD measurements (DOT was not measured, but the authors state that this would be feasible). Oxygen was generated electrochemically in each culture and agitation was provided by a stainless steel bead which mixed the culture, dispersing oxygen and breaking up surface foam. However, this research team provided no comparative bench-scale data with which to determine if scale-up would be feasible from such a device.

Another commercial system for HT operation has been developed by Bioprocessors Corp. (Woburn, MA, USA). This cell cultivation device (called SimCell^®^) is able to operate and independently control up to 1500 cultures thus allowing the use of full factorial experimental design methods for process optimization [60]. This 'reactor-on-a-chip' device is based on a microfluidic design with a gas-permeable membrane enabling oxygen transfer and mixing is provided by rotating the micro-bioreactor array chips in environmentally-controlled incubators using humidified air to minimise evaporation. This system can be highly automated and is integrated with a robot for transferring plates from an incubator to a sensing station for measurement of pH, DOT and cell density and a fluidics station where additions of media for fed-batch operation and acid/base for pH control can be made. Volumes in each reactor range from ca. 300 μl to ca. 700 μl depending on the application (microbial or mammalian cells) and each reactor can be operated in batch, fed-batch or perfusion mode. The device has been shown to support *E. coli *and yeast cultivations, giving growth kinetics comparable to those obtained using conventional STRs. The company has also described growth of CHO cells without oxygen limitation at high cell density and used computational fluid dynamics (CFD) simulations to show how the physical environment seen in large-scale pitched-blade bioreactors has been re-created. k_L_a in the system has been modelled by CFD and estimated at between 60 and 500 h^-1^, values similar to those found in shake flasks and sub-optimal STRs [61].

### MBRs as a scale-down tool

It should be noted that not all miniature cell-cultivation systems are designed for scale-up/scale-down of existing bioprocesses; it has been mentioned in this review how such devices can be used for many applications such as early stage recombinant/wild-type organism appraisal, strain improvement and growth medium development. However, the miniature systems used in the later stages of process development e.g. for optimisation of operation and culture conditions should be scaleable. For this reason it is vital that well established "rule of thumb" methods frequently used in industry to scale from bench-top processes to production vessels are explored to see if they can be utilised to scale-up from MBRs [62]. These proven methods include scaling on the basis of gassed power per unit volume; agitator tip speed; constant DOT; oxygen mass transfer capacity (k_L_a); or mixing time. Yet there is no "one size fits all" approach and so it should be stressed that no single basis for equivalence can be universally applied to all MBRs. None of the systems detailed in this review could utilise all of the established scale-up/scale-down methodologies described above. For example, a constant DOT value is difficult to achieve in shaken systems compared to conventional STRs, as the lack of mechanical agitation (and sparging – in the case of MTP-based systems) mean that control of DOT levels above a critical level in these devices is technically very challenging. This particular feature is not in itself a problem so long as the cells cultivated are sufficiently slow growing (either naturally or through the use of a weak growth medium and/or operating at a temperature not conducive to maximum growth rate), but it does restrict the use of such systems to perform many high cell density processes involving fast-growing microorganisms with high oxygen demand.

An indication of which scale-down criterion should be used for a particular bioprocess (and therefore an indication of which miniaturisation platform is preferable for that process) can be gained by examining the cell characteristics and process conditions of the bioprocess in question. For a fast-growing organism such as *E. coli *or *Bacillus subtilis *it is usually oxygen transfer that becomes limiting, whereas shear stress is not likely to be a major issue; therefore scale-down of such a cell cultivation could be designed on the basis of equal specific power input, or on the basis of equal k_L_a. However, a requirement of choosing equal k_L_a is being able to estimate power input to the miniature bioreactor accurately. Work carried out at UCL in a 10 ml MBR [27] confirms earlier work by Bujalski *et al*. [63] that showed impeller power number to decrease concomitant with vessel diameter. Therefore it is important not to use conventional-scale impeller power numbers for power input estimation in MBRs, as this could lead to oxygen limitation of quickly-respiring microbes by overestimating the power transferred to the system.

A particular challenge is the growth of filamentous organisms due to their complex morphology. Fermentation broths containing such organisms have a relatively high viscosity and require extra power input in order to maintain adequate mixing and mass transfer. In addition, filamentous organisms are much larger than unicellular bacteria and can be more susceptible to shear damage. For example Heydarian *et al*. reported that the average hyphal length of the erythromycin-producing bacterium *Saccharopolyspora erythraea *exceeded the Kolmogorov microscale of turbulence in a standard 7 L bioreactor over a large range of operating conditions [64]. In the case of *S. erythraea *it has been shown that if the mycelia are excessively sheared, resulting in too short a hyphal length, then erythromycin product formation can be affected [65]. For this reason it may be advisable to choose tip speed as the basis for scale-down when using filamentous organisms. Whilst the mechanisms governing pellet formation in filamentous cultures are not well understood, Vecht *et al*. have reported a correlation between decreasing OTR and a reduction in mean pellet size in *Streptomyces tendae *[66]. They concluded pellet formation in that organism is mainly due to hydrophobic interactions controlled by DOT. Given the detrimental effect pellet formation can have on production of secondary metabolites in many filamentous organisms – due to inhibition of oxygen uptake into the centre of the pellet increasing with pellet diameter [65] – it is clear that for the scale down of filamentous cell cultivation processes, MBRs must maintain dissolved oxygen levels found in the large scale process upon which the scale down is based in order to maintain product yield. Equal k_L_a is difficult to use for scale-down as it is usually calculated in model systems that bear little resemblance to actual fermentation broths. Furthermore, k_L_a is affected by changes in culture broth coalescence and rheology over the course of a cultivation process – changes that are very difficult to measure and account for. The key when choosing a basis for bioreactor scale-down is to not expose the cells to stresses over and above those encountered at large scale.

Of the miniature devices discussed in this review, it is clear that some seek to replicate large-scale bioreactors in their geometries. For example, most MSBRs and MBCRs are geometric facsimiles of large-scale bioreactors. Maintaining geometric similarity has advantages for effective scale comparison as it allows some key assumptions to remain valid; e.g. maintaining an equal aspect ratio helps to predict hydrostatic pressure and therefore oxygen solubility at different scales of operation. This gives such devices a benefit as their mechanisms for achieving oxygen transfer and mixing and for calculating power input can be based on the same principles established at large scale. Fluid dynamics will be similar, although it is important to note that some dimensionless numbers describing fluid dynamics, for example Reynolds number in stirred vessels, appear to have less influence at such small scales [67]. More fundamentally there is a question of how effective MBRs can be when they reach such a small size that their flow properties and mechanisms for mass transfer and mixing are different to those found in the large-scale bioreactors they are attempting to mimic. MTPs are particularly vulnerable in this regard as their lack of mechanical agitation means that surface tension effects are more important than in MSBRs, where impellers can decrease this effect and help maintain effective fluid mixing. Furthermore, there is a danger when using extreme conditions with MTPs (in terms of shaking frequency and fill volume) that all of the process liquid forms a thin film along the interior surface of the well, thereby severely limiting mixing and exacerbating the detrimental effect of surface tension. Different flow regimes in MBRs caused by different methods of agitation can have an impact on the ability of such systems to reproducibly perform cell cultivations; if conditions are different at small and large scale in terms of mixing and gas-liquid mass transfer this could lead to problems, e.g. the selection of clones not suitable for production or differences in the product quality especially for recombinant proteins. On the other hand, work by Micheletti *et al*. indicates that scale translation from shaken to stirred systems is feasible if scale-up criteria are carefully chosen [68]. Using a recently introduced correlation for k_L_a predictions in MTPs [69] they were able to successfully scale up the cultivation of *E. coli *over-expressing a transketolase enzyme from a microwell system (1 mL volume) to a 1.4 L STR on the basis of constant k_L_a. The same group also provide initial data on satisfactory scale-up of a mammalian cell culture process using a constant mean energy dissipation rate [68].

### Automation of MBRs

The automation of MBRs is the key to expanding HT capability. Several of the miniature systems recently developed utilise a modified MTP as their starting point (e.g. [49,59] and the Applikon MicroReactor^®^). These systems currently appear to offer a great deal of promise due to their ease of integration with existing robotic automation platforms. MTPs on which such systems are designed are based on a standard footprint, are mechanically-simple and the very standardisation of their design makes them ideal to build into automated, robotic platforms that truly take such technologies into the HT domain, conferring upon them the ability to perform hundreds of cell-cultivations in parallel, using a footprint not much larger than that of a conventional pilot-scale bioreactor. The alternative is to develop a miniature bioreactor system that is itself amenable to automation. The technologies that Weuster-Botz's group in collaboration with H + P Labortechnik [43,44] and Bioprocessors Corp. have developed are examples of this approach. Such devices offer a degree of HT capability as well as sophisticated in-built robotics in the case of Bioprocessors Corp's SimCell^® ^system.

Robotic devices used in conjunction with MBRs usually feature multiple-pipetting heads mounted on arms that are able to move in three dimensions across the entire working area. The pipetting heads may also cope with different MBR geometries and separate robotic arms can pick and place ancillary equipment anywhere in the work space. This pick and place ability means that one robot can inoculate, pH-control, sample and make additions to an MBR, offering a truly integrated solution. In addition robots can link cell cultivation platforms with analytical instruments (e.g. HPLC systems) and perform complex assays such as ELISA for antibody products using real-time samples – assays that take advantage of the robot's ability to perform thousands of liquid-handling operations in a short period of time. Aseptic cell-cultivation conditions can be maintained by housing the robot within a custom-built biosafety cabinet.

## Conclusion

This review has described many of the approaches currently being taken towards the development of MBRs and has highlighted several challenges to overcome in the design of such systems. Most of these stem from the variability of the tasks having to be performed, which makes it difficult to find a single system able to satisfy all requirements. For example, if using a miniature platform for growth medium development, a need for parallelism would take precedence, whereas detailed strain characterisation and optimisation of process conditions would require a higher degree of instrumentation of each bioreactor. In this instance, HT capability would not be paramount. Furthermore, devices that agitate cultures through shaking (e.g. MTPs and shake flasks) typically exhibit a lower OTR capability relative to MSBRs, making these mechanically-agitated devices most promising for the cultivation of fast-growing microorganisms or cell cultivations that reach a high-cell density; however, there is typically not the same degree of parallelism available in MSBRs. Although both MSBRs and MTPs are able to be integrated with robotic systems, the fact that most existing liquid handling robots are build to handle plates means it is likely to be those devices that base their design upon an MTP that will be able to offer the highest throughput.

In view of the development of whole bioprocess sequences on the deck of a robot as first proposed by Lye *et al*. [5] and recently demonstrated for three steps of a biocatalytic process [70], one must also consider how MBRs integrate with miniaturised downstream processing technologies. This is of particular relevance when MBRs are used as development tools for the production of biomolecules such as recombinant proteins that have to be subsequently purified and analysed in order to assess their biological activity. In this respect, Jackson *et al*. have developed a prototype miniature microfiltration device that performs several operations simultaneously [71]. There is a danger of being able to carry out hundreds of simultaneous cell-cultivations yet not having complementary technology developed for product recovery and purification; it is the opinion of the authors that for applications where further processing of the culture (broth or cells) is required, some current miniaturised systems employ culture volumes that may be too small for evaluation of subsequent steps.

The key message of this review is that there is no single MBR that satisfies all requirements equally. Primarily this is because there is a need to differentiate microscale systems and the advantages that each one confers depending on the application. Therefore one may use different systems for different stages of the development process taking into account the nature of the cells and the complexity of the cultivation process – especially the requirements for monitoring, control and sampling.

## Competing interests

The author(s) declare that they have no competing interests.

## Authors' contributions

JB carried out the literature review and drafted the manuscript. FB critically reviewed and edited the manuscript. Both authors read and approved the final manuscript.
